# The Birth-Rate in New South Wales

**Published:** 1904-12-03

**Authors:** 


### THE BIRTH-RATE IN NEW SOUTH WALES.

At the annual meeting of the Sanitary Inspectors' Association, held at Carpenters' Hall on Oct. 29, attention was called to the progress of sanitation in New South Wales, and, more especially, to the recently issued report of a Royal Commission on the decline of the birth-rate and the mortality of infants in that colony. This report showed that between 1880 and 1888 the birth-rate had remained almost stationary at about 38 per 1,000 living, but that in 1889, the year of the diffusion of Neo-Malthusian literature in the colony, a sudden drop took place, and since then there has been a gradual decline until 1902, when the rate stood at 27·7. The fall in birth-rate, together with the excess of mortality amongst illegitimate over legitimate children, has resulted in a loss of population in 38 years of 250,000 in New South Wales, and of 940,000 in Australia generally. The Commissioners are unable to attribute the lessened fertility to any natural cause, and say that it must be traced to the operation of a force or forces over which the people themselves have control. The evidence leaves them no option but to attribute it to deliberate prevention. Practices to secure this object are, they affirm, common among all classes of the community, have greatly increased during the last fifteen years, and have led to the development of an unwholesome trade of large volume. They believe that the limitation of families is in a certain number of instances due to a conviction that a want of adequate means for the rearing of children justifies interference with the course of nature, but in a large majority of instances it is dictated by pure selfishness. It is an unwillingness to submit to the strain and worry of children, a desire to avoid the discomfort of childbearing, and a love of luxury and social pleasures that has led to the adoption of checks on reproduction that threaten the economic welfare of the colony, and often entail serious injury to bodily health, to say nothing of moral decadence, in those who avail themselves of them. The medical evidence is overwhelming that these checks are responsible for some loss of life through blood-poisoning, numerous cases of disease of the pelvic viscera, much permanent sterility, and a great increase in neurasthenia and insanity; while clerical witnesses testify that they are destructive of the sanctity of marriage, lower the standard of right thinking and right living in the community, create laxity of morals, and debase character. The Commissioners, after suggesting certain legal palliatives, declare that the real want of the colony is a revival of moral sanitation. They say that the people in New South Wales, "led astray by false and pernicious doctrine into the belief that personal interests and ambitions, and a high standard of ease, comfort, and luxury are the essential aims of life, and that these aims are best attained by refusing to accept the consequences which nature has ordained shall follow from marriage, have neglected and are neglecting their duty to themselves, to their fellow countrymen, and to posterity. Forgetful of the teachings of history, ignoring the teachings of science, they seem to think that in the deliberate curtailment of reproduction they have found a panacea for all the ills of life. The time must come, however, when there must be a cruel awakening to a realisation of the truth. Already we see in the injury to health and in the wrecking of life which are manifesting themselves, how nature has begun to avenge herself on those who oppose her laws. We see in the lessening of parental control the commencement of dissolution of the family bond, and, in the dwindling of the size of families, the dying out of nature's best school for teaching the lessons of life." We feel that the thanks, not only of the colony concerned, but of the whole civilised world, are due to the authors of this wise and courageous document, and we can only hope that the whole of the report will soon be rendered readily accessible in this and in other countries.

